# EBER-negative inflammatory pseudotumor-like follicular dendritic cell sarcoma of liver: A case report

**DOI:** 10.1097/MD.0000000000037651

**Published:** 2024-04-05

**Authors:** Qiang Zhang, Jialin Gao

**Affiliations:** a Department of Hepatobiliary Surgery, Affiliated Hospital of Xuzhou Medical University, Xuzhou, Jiangsu Province, China; b Department of Pathology, Affiliated Hospital of Xuzhou Medical University, Xuzhou, Jiangsu Province, China.

**Keywords:** Epstein-Barr virus, inflammatory pseudotumor, like follicular cell sarcoma, liver tumor

## Abstract

**Rationale:**

Inflammatory pseudotumor-like follicular dendritic cell sarcoma (IPT-like FDCS) of the liver is rare. It was previously believed that Epstein-Barr virus (EBV) positivity was a necessary criterion for pathological diagnosis. However, we found that there were also cases of EBV negativity. Therefore, clinicians and pathologists are reminded that EBV positivity is not a necessary condition for diagnosis.

**Patient concerns:**

A 70-year-old female underwent computed tomography (CT) examination for upper abdominal discomfort, which revealed the presence of a liver tumor. Follow-up revealed that the tumor had progressively increased in size.

**Diagnosis:**

The final diagnosis was an IPT-like follicular cell sarcoma, based on CT, MRI, HE staining, and immunohistochemical staining.

**Interventions:**

The patient underwent a laparoscopic left hemihepatectomy.

**Outcomes:**

The patient has not undergone any special treatment, such as radiotherapy and chemotherapy, and has been followed up for over 3 years without experiencing any recurrence.

**Lessons:**

IPT-like FDCS is a rare tumor that lacks definitive criteria, and its diagnosis mainly relies on pathological findings. Previously, it was believed that being EBV-positive was an important condition for diagnosis. Primary IPT-like FDCS in the liver is even rarer, and the patient in this case tested negative for EBV. It may be necessary for pathologists to consider the role of EBV in the diagnosis of IPT-like FDCS.

## 1. Introduction

Follicular dendritic cell sarcoma (FDCS) is a rare malignant tumor that exhibits characteristics of differentiation into follicular dendritic cells.^[1]^ In 1986, Monda^[2]^ first described it in a report of 4 cases of lymphadenopathy. Inflammatory pseudotumor (IPT)-like FDCS is a rare subtype of FDCS, with fewer than 100 reported cases worldwide. It is more common in women and occurs in the liver and spleen. Tumor cells of IPT-like FDCS are rare and scattered in a significant inflammatory background consisting of a large number of lymphocytes, plasma cells, and histiocytes, which closely resemble an IPT.^[3]^ Currently, more than 95% of the reported cases of IPT-like both domestically and internationally are found to be co-infected with Epstein-Barr virus (EBV). Only a small number of cases occurring in the colon, ileum, mesenteric, axillary, and cervical lymph nodes have been reported as EBER-negative.^[4–6]^ Here, we report a case of EBER-negative IPT-like of the liver, which is the fourth case reported worldwide, according to a literature review.^[7]^

## 2. Case presentation

**Figure 1.**
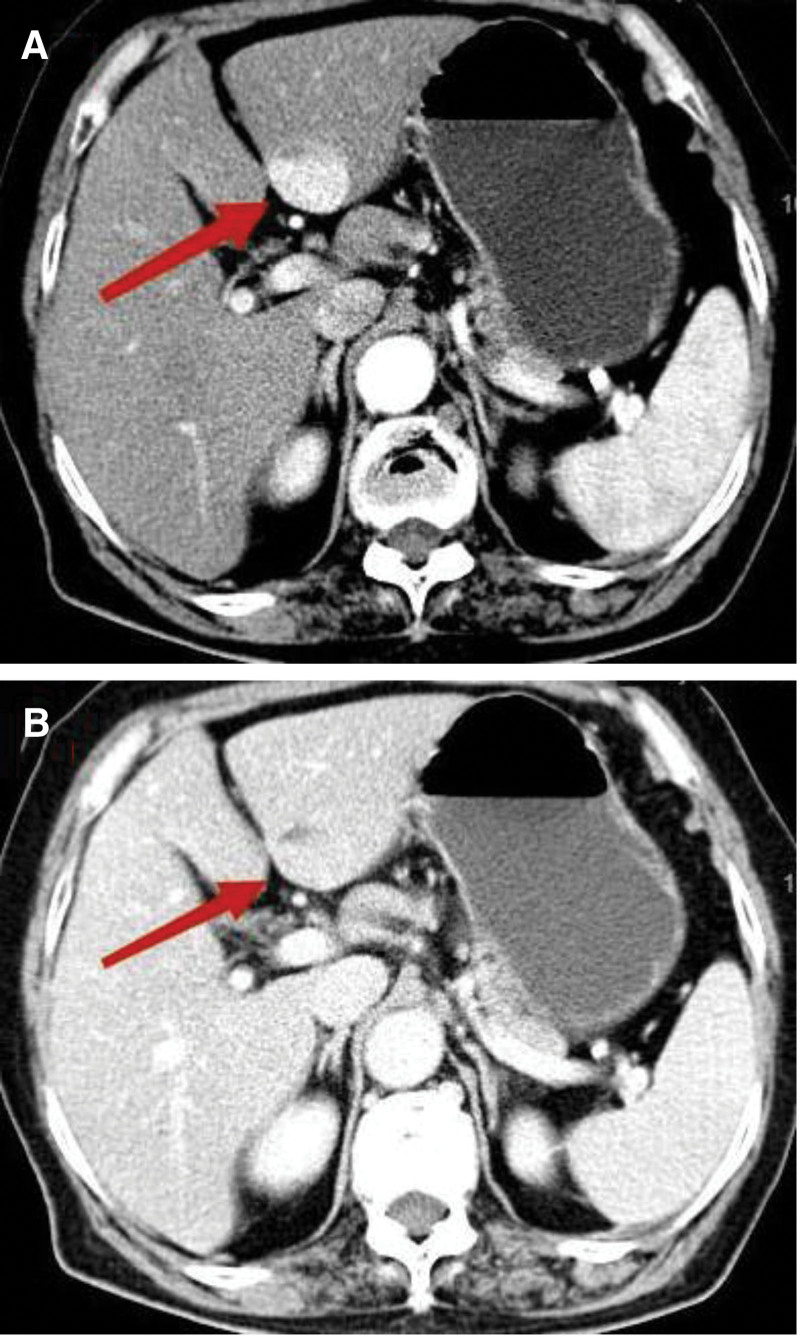
(A) CT contrast-enhanced arterial phase shows significant enhancement of the left lateral lobe of the liver, with patchy suspicious fatty density; (B) venous phase shows similar enhancement to the liver parenchyma. CT = computed tomography.

**Figure 2.**
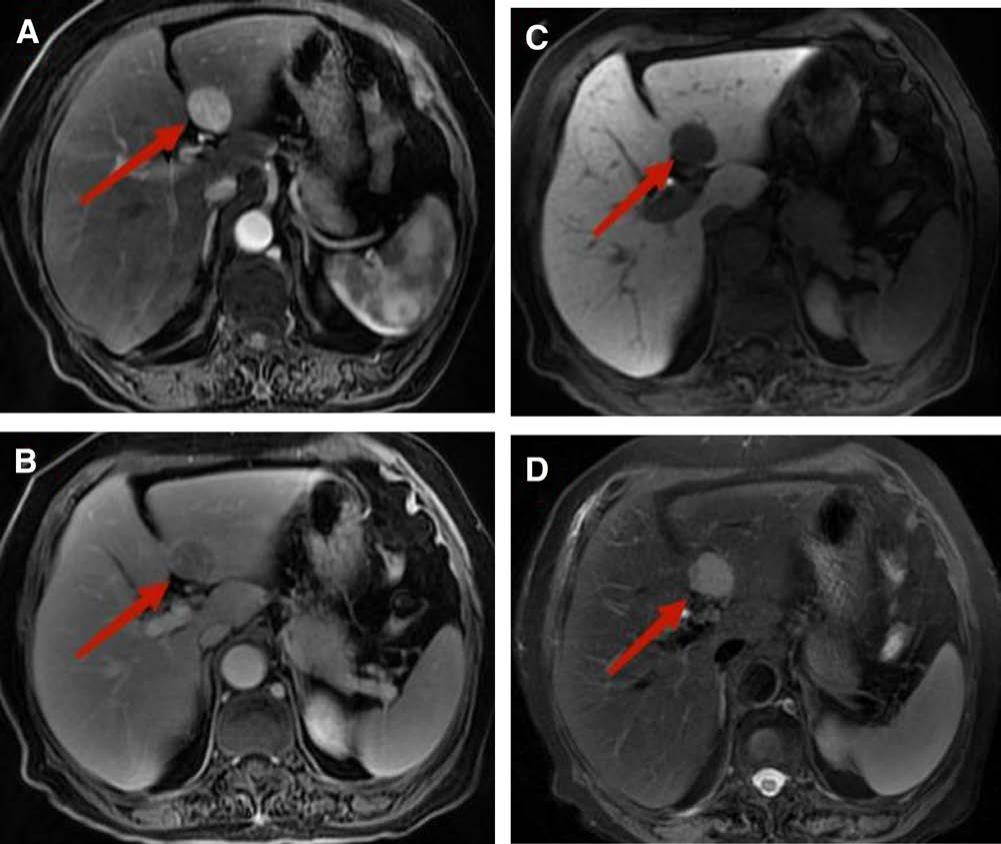
(A) Liver MRI with disodium gadolinite showing significant enhancement during the arterial phase; (B) Lesion signal is lower than liver signal during the venous phase; (C) Low signal during the excretory phase; (D) Long signal decay during the T2 phase.

A 70-year-old female was admitted to the hospital due to dull pain in the right upper abdomen. One year prior, the patient had a space-occupying lesion measuring 29 × 25 mm in the left lateral lobe of the liver, which was detected on an enhanced computed tomography (CT) scan. Additionally, the patient was diagnosed with gallstones and cholecystitis at that time. Enhanced CT showed significant enhancement of the lesion during the arterial phase, with a degree of enhancement similar to that of the liver parenchyma during the venous phase. There was a patchy, suspicious fatty density shadow within the lesion, suggesting a hepatic angiomyolipoma (Fig. 1). Further improvement of the MRI with disodium gadolinite showed that the S3 segment of the liver exhibited significant enhancement during the arterial phase, decreased enhancement during the venous phase, and low signal during the excretory phase, suggesting hepatocellular carcinoma (Fig. 2). The patient had a history of thyroid cancer and had recovered well after the surgery. She took levothyroxine tablets for an extended period after the surgery. After admission, the patient underwent further laboratory tests, including alpha-fetoprotein: 3.89 ng/mL, PIVKA-II: 20 mAu/mL, and CA19-9: 5.9 U/mL, AST: 9 U/L, ALT: 9 U/L, GGT: 29 U/L, ALP: 131 U/L, and TBil: 9 µmol/L. After admission, a CT scan showed that the tumor had significantly increased in size compared to the previous scans, measuring 39 × 42 mm. The patient underwent a laparoscopic left hemihepatectomy, recovered smoothly after the surgery, and was discharged.

**Figure 3.**
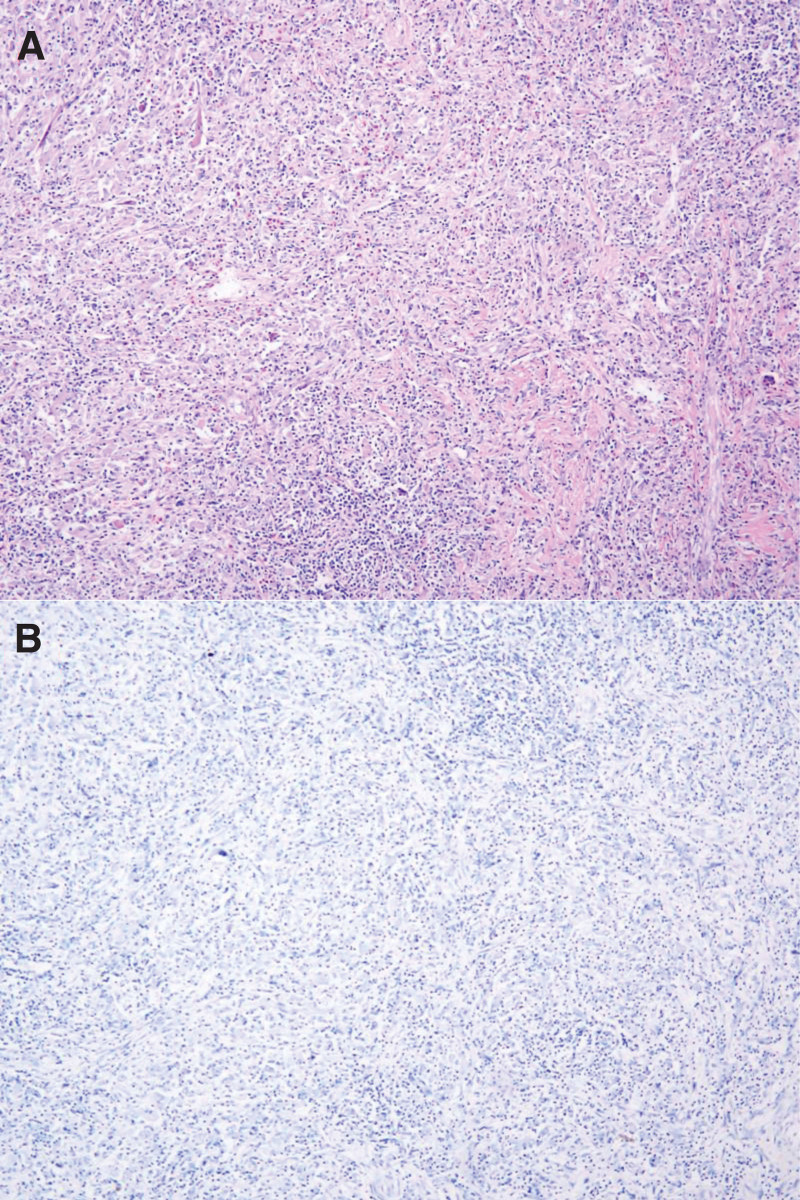
(A) Large areas of lymphoid tissue and fibroblasts within the tumor stained with HE; (B) EBER in situ hybridization test negative.

The tumor in the patient postoperative specimen exhibited a distinct demarcation from the surrounding liver, characterized by a grayish-yellow cut surface and a moderate texture. HE staining revealed large areas of proliferating lymphoid tissue and scattered large cells with prominent nucleoli, large nuclei, and abundant red-stained cytoplasm in the tumor. Immunohistochemical results showed the following: tumor cells were positive for CD35, D2-40, Clusterin, Fascin, CD68, CD163, CD21, and GS; negative for CD30, EGFR, S100, HMB45, ALK, SMA, Desmin, CK, Hepa, Glypican-3; Ki-67 was 5%; EBER was negative. Background cells were positive for LCA, CD20 (small amount), CD3, CD4, CD8, CD38, CD138, KAPPA, LAMBDA (plasma cells), and a few cells were positive for IgG4 (Fig. 3).

The patient did not receive any special treatment after the operation and showed no signs of recurrence during the 3-year follow-up period.

## 3. Discussion

In 1996, Arber^[8]^ found that 41.2% (7 out of 18) of the IPT specimens contained EBV-positive spindle or oval cells. These cells were also found to express SMA, Vimentin, or FDC-related markers. Through successive reports, researchers gradually realized that some IPTs were accompanied by the proliferation of follicular dendritic cells during EBV infection. Arber believed that these lesions belong to a distinct subtype of IPT, which could potentially result in the formation of FDCS-related tumors. In 2001, Cheuk^[9]^ analyzed 11 such cases and proposed the term “IPT-like FDCS” to name this special lesion for the first time. They believed that this particular subtype of FDCS had distinct clinical and pathological characteristics, making it a unique subtype of FDCS. Compared to the classic type, IPT-like FDCS is rarer, exhibiting significant infiltration of mixed inflammatory cells in the tumor background. The number of spindle tumor cells is sparse and scattered, which is very similar to that of the IPT. Owing to the lack of specificity in clinical and laboratory examinations, imaging examinations often reveal a well-defined space-occupying lesion, which can lead to misdiagnosis as an IPT or another spindle cell proliferative tumor. Therefore, it is difficult to distinguish between the 2 based on morphology alone, and immunohistochemical staining is necessary. The majority of IPT-like FDCS express one or more FDC markers, with the most commonly used markers being CD21, CD23, or CD35. These markers can be used as first-line antibodies. In contrast, IPTs only show focal positive expression in a few cases.

EBER in situ hybridization plays an important role in the diagnosis of IPT-like FDCS, as almost all of these tumors are associated with EBV infection. Therefore, pathologists believe that EBER in situ hybridization testing plays a more important role in diagnosing IPT-like FDCS. Almost the vast majority of these tumors are associated with EBV infection, with only a few cases of EBV-negative IPT-like FDCS occurring in the colon, ileum, and mesentery.^[4,5]^ They believe that such EBV-related IPTs, regardless of whether they express FDC markers or not, should be given the same clinical evaluation, treatment, and follow-up as IPT-like FDCS.

EBV is a common carcinogenic factor in clinical practice, and infection often causes damage to multiple organs throughout the body, with liver damage being the most common.^[10]^ Additionally, the EBER in situ hybridization test shows mostly positive results. Southern blot hybridization can also be used to detect EBV antibody-free genes. Horiguchi^[11]^ also found, through DNA sequencing, that tumor cells infected with EBV expressed the latent membrane protein-1 gene with a 30-bp sequence deletion and 3-point mutations. Although the specific mechanism has not been clarified, some studies have shown that the EBV gene establishes latent infection by integrating into the host cell genome. The expression of viral products then drives cell proliferation, ultimately leading to tumorigenesis.^[12]^

Currently, there are no uniform guidelines or recommendations for the treatment of IPT-like FDCS. However, surgical resection has generally been agreed upon as the preferred treatment. In this case, the patient underwent surgical resection and has been followed up without any recurrence to date. There is no research to support the need for adjuvant chemotherapy or radiotherapy after surgery, and most treatment regimens involve long-term follow-up after surgery.^[13,14]^

## 4. Conclusion

Currently, testing for EBV positivity is considered the primary diagnostic method for IPT-like FDCS. However, through this case, EBV infection may not be necessary for diagnosing IPT-like FDCS. Due to the small and scattered number of reported cases, the current understanding of its clinical characteristics, diagnosis, prognosis, and treatment is still quite limited.

## Acknowledgments

We would like to thank the patient and her family.

## Author contributions

**Conceptualization:** Qiang Zhang.

**Data curation:** Qiang Zhang.

**Formal analysis:** Qiang Zhang.

**Resources:** Jialin Gao.

**Supervision:** Qiang Zhang.

**Validation:** Jialin Gao.

**Writing – original draft:** Qiang Zhang.

**Writing – review & editing:** Qiang Zhang, Jialin Gao.
